# The Dead Weight of the Sick

**Published:** 1896-02-01

**Authors:** 


### The Hospital

An Institutional Journal of

The Medical Sciences and Hospital Administration, with

Vol. XIX.—No. 488.] AN EXTRA NURSING SUPPLEMENT. [Feb. 1, 1896.

### The Dead Weight of the Sick.

Public health means public capacity and public effectiveness. Notwithstanding many years of diligent instruction on the part of medical statesmen, large numbers, even of rational persons, still persist in seeing nothing else in our vast masses of sick people but a horde of helpless creatures who need relief. That the sick are helpless and need relief is quite true; it is also true that an army is often hungry and needs to be fed. But nobody affirms that the proper business of an army is to eat, or that the sole duty of the taxpayer is to feed it. An army is for fighting, and its feeding is but an incident. So likewise the business of every member of the community is to do his life's work; and the man throughout his life must be looked at from the point of view of his working capacity. Any sickness he may have to encounter is but an incident. It is, however, very often a costly incident both to himself and to the community, and it is, therefore, the business of the community, as a community, to do everything which science can teach to prevent the incident of sickness altogether if possible, and, where that is not possible, to reduce the period of ineffectiveness from it to a minimum. That these propositions are both scientific and logical nobody denies. Yet when it is proposed to deduce from them several conclusions to be carried into practical effect, forthwith they are condemned, even by so able a person as Lord Thring, as mere "counsels of perfection" which cannot by any possibility be reduced to practice.

This little excursus into the region of general principles is *apropos* of a paper read by Dr. Arthur Newsholme the other evening before the Royal Statistical Society, on the "Notification and Registration of Sickness." Dr. Newsholme is of opinion that it would be a great advantage to the community if it could have all its sick members, with their various preventable and non-preventable illnesses, systematically pictured before it, so that it could, whenever it pleased, and for practical purposes, take an intelligent bird's-eye view of the whole situation. Such a full, intelligent, and systematic picturing of the sick and their preventable and non-preventable ailments, Dr. Newsholme thinks, could be effectively secured by means of an Imperial system of "notification and registration."

Certain diseases, the so-called zymotic diseases, are already notified. Some advantages have resulted from their notification, and many more will follow. Deaths, all deaths, are registered; and an account, more or less full and true, is given of their causes. The registration of deaths the community finds to be not only desirable but indispensable. It is one of the "resources of civilisation." We are at one with Dr. Newsholme in thinking that more advantages would follow from the notification and registration of all classes of sick persons and their ailments than has resulted from the systematic registration of deaths. There are here two things to be considered, the death-rate and the health-rate. Which is the more important of the two? It has been usual to consider the two synonymous. That is by no means a true view of the case. Mortality is one thing; morbidity is another and quite a different thing. When a man is dead there is no more to be said; when he is merely ill there may be very much more to be both said and done; when he is quite well there is most of all to be said and done in order that, if possible, he may be kept quite well.

It should be constantly borne in mind that medical men, in reasoning as we are now reasoning, are not theorising. They are doing their utmost to proclaim, as from the housetops, the facts and doctrines of a physical religion which they have over and over again tested and verified in their own experience. No person, indeed, is so little given to mere theorising as the modern medical man. Experiment, experience, trying, and proving—these are his watchwords from day to day and from year to year. Even when he has tried and proved, he can only be persuaded with the greatest possible difficulty to make efforts to get his science and his truth embodied in the laws and life of the community. The community is so unscientific, he argues, so dull in taking up new ideas, so prejudiced against medical men, so given over to making money, or making war, or making love, that for the great and beneficent discoveries and deductions of science it has neither eyes nor ears, unless they happen to be as big as mountains and as obvious as the moon or the sun.

The sick of the community are a dead weight, most distressing, most costly. The dead weight can be lightened immensely; the cost can be indefinitely reduced. If anyone asks, "Where is the proof of this?" the answer is that proof upon proof can be supplied by the experience of small communities. Take Worthing as a case in point. Quite recently that town was decimated and half ruined, financially, by an attack of illness of a kind which was as preventable as a wetting is preventable by keeping indoors in a heavy shower of rain. Small communities—health resorts and other similar places—although they do not notify and register all their cases of sickness, yet in practice keep such a watchful eye upon the health of their people, from mere motives of self-interest, that their death-rate is often less than half the death-rate of communities which have no immediate financial interest in preserving the general health. That which is true of small communities is true in a fuller, larger, and far more costly sense of the large community which we call the nation. Dr. Newsholme's argument in favour of the notification and registration of sickness may for the present moment be a mere "counsel of perfection"; but it is a counsel which any nation that lays claim to science and civilisation will take care to convert into a "counsel of practice" with as little delay as may be possible.

